# The Effect of Statin on Epithelial-Mesenchymal Transition in Peritoneal Mesothelial Cells

**DOI:** 10.1371/journal.pone.0109628

**Published:** 2014-10-02

**Authors:** Tae Ik Chang, Hye-Young Kang, Kyung Sik Kim, Sun Ha Lee, Bo Young Nam, Jisun Paeng, Seonghun Kim, Jung Tak Park, Tae-Hyun Yoo, Shin-Wook Kang, Seung Hyeok Han

**Affiliations:** 1 Department of Internal Medicine, College of Medicine, Brain Korea 21 Project for Medical Sciences, Severance Biomedical Science Institute, Yonsei University, Seoul, Korea; 2 Department of Internal Medicine, NHIS Medical Center, Ilsan Hospital, Goyang-shi, Gyeonggi-do, Korea; 3 Department of Surgery, College of Medicine, Brain Korea 21 Project for Medical Sciences, Yonsei University, Seoul, Korea; Seoul National University, Republic of Korea

## Abstract

**Background:**

Statins have recently been highlighted for their pleiotropic actions distinct from cholesterol-lowering effects. Despite this interest, it is currently unknown whether statin therapy inhibits peritoneal dialysis (PD)-related epithelial-mesenchymal transition (EMT).

**Methods:**

*In vitro*, human peritoneal mesothelial cells (HPMCs) were exposed to 5.6 mM glucose (NG) or 100 mM glucose (HG) with or without simvastatin (1 µM). *In vivo*, PD catheters were inserted into 32 Sprague-Dawley rats, and saline (C, n = 16) or 4.25% peritoneal dialysis fluid (PDF) (PD, n = 16) was infused for 4 weeks. Eight rats from each group were treated with 5 mg/kg/day of simvastatin intraperitoneally. Changes in the protein expression of EMT markers such as E-cadherin, α-SMA, Snail, and fibronectin in HPMCs and the peritoneum were evaluated by Western blot analysis and immunofluorescence or immunohistochemical staining. We also explored whether activation of the mevalonate pathway and its downstream small GTPases were involved in dialysis-related peritoneal EMT and could be inhibited by statin treatment.

**Results:**

Compared to NG cells, E-cadherin expression was significantly decreased, while α-SMA, Snail, and fibronectin expression were significantly increased in HPMCs exposed to HG, and these changes were abrogated by simvastatin (p<0.05). In addition, the cobblestone-like appearance of normal HPMCs was converted into a fibroblast-like morphology after HG treatment, which was reversed by simvastatin. These EMT-like changes were also observed in HPMCs treated with geranyl-geranyl pyrophosphate (5 µM). HG significantly increased the protein expression of RhoA and Rac1 in the membrane fractions, and these increases were ameliorated by simvastatin (p<0.05). In PD rats, E-cadherin in the peritoneum was significantly decreased, whereas α-SMA, Snail, and fibronectin expression were significantly increased (p<0.05) compared to C rats. The thickness of the mesothelial layer in the peritoneum were also significantly greater in PD rats than in C rats (p<0.05). These changes of the peritoneum in PD rats were significantly attenuated by simvastatin.

**Conclusion:**

This study demonstrated that PD-related EMT was mediated via the mevalonate pathway, and statin treatment inhibited the EMT changes in HG-treated HPMCs and PDF-stimulated PD rats. These findings suggest that statins may be a promising therapeutic strategy for preservation of peritoneal membrane integrity in long-term PD patients.

## Introduction

Even though peritoneal dialysis (PD) is generally accepted as an established modality for the management of patients with end-stage renal disease (ESRD), a concern about peritoneal membrane failure has consistently been raised in long-term PD. Many factors have been demonstrated to be involved in the development of peritoneal dysfunction. In particular, the nonphysiologic nature of PD solutions—high concentrations of glucose and lactate, low pH, and glucose degradation products—is a major factor responsible for deleterious effects on the peritoneal membrane [1], [2]. These components also induce chronic inflammation in the peritoneal cavity, which is often exacerbated by recurrent episodes of peritonitis and consequently leads to structural and functional alterations of the peritoneal membrane [3].

Peritoneal fibrosis (PF) is the ultimate form of peritoneal damage. It is characterized by the loss of the peritoneal mesothelial cell (PMC) monolayer, submesothelial fibrosis, angiogenesis, and hyalinizing vasculopathy [3]–[6]. In the past, resident stromal fibroblasts and inflammatory cells had been considered to be the main cells responsible for PF [7], [8]. Recently, however, PMCs have emerged as an active player in the alteration of the peritoneal membrane. After PD initiation, PMCs progressively lose their epithelial characteristics and acquire a myofibroblast-like phenotype through the process of epithelial-mesenchymal transition (EMT) [5]. EMT is a normal physiologic process during embryo implantation, embryogenesis, or organ development, but it is also involved in various pathologic processes, including cancer metastasis and fibrotic disorders [7]. Indeed, EMT enables PMCs to gain migratory and invasive capacities, thus they can intrude into the subepithelial stroma and produce extracellular matrix (ECM) components such as fibronectin and collagen, which ultimately leads to PF [8].

Recently, numerous aspects of 3-hydroxy-3-methyl-glutaryl-coenzyme A (HMG-CoA) reductase inhibitors, or statins, have been highlighted due to their pleiotropic effects aside from their lipid-lowering property [9]–[11]. Of note, one of the key actions of statins is inhibition of the downstream products of the mevalonate pathway such as farnesyl pyrophosphate (FPP) and geranyl-geranyl pyrophosphate (GGPP) [12]. As a result, isoprenylation of small RhoGTPases and Ras, the final products of this pathway, is inhibited [13]–[15]. Interestingly, previous studies have found that activation of small RhoGTPases such as RhoA, Rac1, and Cdc42 plays a key role in the process of EMT implicated in diverse renal diseases [16]–[19]. In addition, a recent study by Zhang *et al.* [20] showed that the RhoA/ROCK signaling pathway mediated EMT in rat PMCs in response to transforming growth factor (TGF)-β1. These findings suggest that statins may reverse EMT-like changes through the inhibition of isoprenylation of small RhoGTPases. However, to our knowledge, this assumption has not yet been tested. In this study, therefore, we investigated the effect of statins on PD-related EMT both *in vitro* and *in vivo*.

## Materials and Methods

### Ethics statement

This study was carried out in strict accordance with the recommendations in the Guide for the Care and Use of Laboratory Animals of the National Institutes of Health. The protocol for all animal experiments was approved by the Ethics Committee and the Institutional Animal Care and Use Committee of Yonsei University College of Medicine.

Human omental tissue was obtained from patients who underwent elective abdominal surgery. For the use of omental tissue, we obtained written informed consent from these patients and received approval from the Institutional Review Board of our institution.

### Isolation of human PMCs (HPMCs)

HPMCs were isolated according to the method described by Stylianou *et al* [21]. Briefly, a piece of human omentum was washed three times with sterile phosphate-buffered saline (PBS) and incubated in a 0.05% trypsin-0.02% ethylenediaminetetraacetic acid (EDTA) solution for 20 min at 37°C with continuous shaking. After incubation, the suspension containing free HPMCs was centrifuged at 100×g for 10 min at 4°C. The cell pellet was then washed once and re-suspended in M199 medium supplemented with 10% fetal bovine serum (FBS), 100 U/ml penicillin, 100 mg/ml streptomycin, and 26 mM NaHCO_3_, and seeded onto culture dishes. The cells were grown in the same medium at 37°C in humidified 5% CO_2_ in air, and the medium was changed 24 hr after seeding, and then every 3 days.

### HPMCs experiments

Subconfluent HPMCs were serum-restricted for 24 hr, and the medium was then changed to serum-free M199 medium containing normal glucose (5.6 mM, NG), NG + mannitol (94.4 mM, NG+M), NG + simvastatin (1 µM) (Sigma Chemical Co., St Louis, MO, USA), or high glucose (100 mM, HG) with or without simvastatin (1 µM). The dose of simvastatin used in the experiments was determined using a 3-(4,5-dimethylthiazol-2-yl)-2,5-diphenyltetrazolium bromide (MTT) cell viability assay and trypan blue exclusion. To explore whether isoprenoids of the mevalonate pathway were involved in peritoneal EMT, HPMCs were treated with NG+GGPP (5 µM) (Sigma Chemical Co.). HPMCs exposed to HG were also treated with Rho/ROCK inhibitor (Y27632, 1 µM) (Sigma Chemical Co.) or Rac inhibitor (EHT1864, 1 µM) (R&D Systems, Minneapolis, MN, USA). At 72 hr after the media change, cells were harvested.

#### Evaluation of small GTPase activation

To examine the activation of small GTPases, membrane and cytosol proteins were prepared separately and the expression of RhoA and Rac1 were determined in each fraction by Western blotting. Briefly, HPMCs treated as above were washed with cold PBS and lysed by freeze-thawing in ice-cold lysis buffer containing 50 mM HEPES (pH 7.4), 5 mM NaCl, 1 mM MgCl_2_, 2 mM EDTA, 1 mM dithiothreitol, 10 mM sodium fluoride, 1 mM phenylmethylsulfonyl fluoride, 10 µg/ml aprotonin, and 10 µg/ml leupeptin (Sigma Chemical Co.). The homogenates were centrifuged at 4°C and 100,000×g for 30 min and the resulting supernatant (cytosolic fraction) was collected. The pellets were then homogenized in the same lysis buffer containing 2% Triton X-114 and centrifuged at 800×g for 10 min at 4°C, and the supernatant was collected. This supernatant was referred to as the membrane fraction. The activity of Rho-kinase was determined by using the colorimetric G-LISA RhoA activation assay biochemical kit (Cytoskeleton, Denver, CO, USA) according to the manufacturer's protocol as previously described [22].

### Animal studies

Peritoneal access ports were inserted in 32 male Sprague-Dawley rats weighing 250–280 g, and 2 ml of saline with 1 IU/ml heparin was instilled intraperitoneally until wound healing. One week after surgery, 16 rats received a daily (once per day) 20 ml of saline instillation and 16 rats were instilled daily with 20 ml of 4.25% peritoneal dialysis fluid (PDF, Dianeal, Baxter Healthcare Ltd., Singapore) for 4 weeks. Eight rats from each group were treated with simvastatin (5 mg/kg per day) intraperitoneally, while 8 rats in each group were left untreated (control). Simvastatin dose was determined based on the previous studies [23], [24]. After 4 weeks of PD, the abdomen was opened by a midline incision and the entire anterior abdominal wall was removed at the contralateral side to the tip of the implanted catheter. One fifth of the whole tissue adjacent to the liver was fixed in 10% neutral-buffered formalin for pathologic examination, while the parietal peritoneum dissected from the major part of the tissue was washed in ice-cold PBS, snap-frozen in liquid nitrogen, pulverized with a mortar and pestle while frozen, and suspended in SDS sample buffer [2% SDS, 10 mM Tris-HCl, pH 6.8, 10% (vol/vol) glycerol]. After centrifugation at 16,000×g for 15 min at 4°C, the supernatant was kept at −80°C until use.

### Western blot analysis

The protein expression of E-cadherin (BD Biosciences, San Jose, CA, USA), Snail (Abcam, Cambridge, UK), α-SMA (Sigma Chemical Co.), fibronectin (DAKO, Glostrup, Denmark), RhoA (Santa Cruz Biotechnology, Inc., Santa Cruz, CA, USA), and Rac1 (Abcam) in HPMCs and peritoneal tissue was evaluated by Western blot as previously described [25]. The band densities were measured using Image J software v1.60 (National Institutes of Health Image software, Bethesda, Maryland, USA; online at http://rsbweb.nih.gov/ij), and the densitometric intensity corresponding to each band was normalized with α/β tubulin expression. The changes in the optical densities of bands from the treated groups relative to NG cells or the peritoneum of control rats were used in the analysis.

### Immunofluorescence staining

HPMCs grown on chamber slides were fixed in 4% paraformaldehyde for 10 min at 4°C, washed three times with PBS, and incubated with 1% BSA for 20 min at room temperature. For immunofluorescence staining, primary polyclonal antibodies to E-cadherin, Snail, α-SMA, RhoA, and Rac1 were diluted in 1∶100 with antibody diluent (DAKO) and were applied for 3 hr at room temperature. After washing with PBS, Cy3 (red)- or Cy2 (green)-conjugated anti-rabbit IgG antibody (Research Diagnostics, Inc., Flanders, NJ, USA) was added for 60 min.

### Immunohistochemical and Masson's trichrome staining

The peritoneum samples were fixed in 10% neutral-buffered formalin, processed in the standard manner, and 5 µm-thick sections of paraffin-embedded tissues were utilized for immunohistochemical staining. Slides were deparaffinized, hydrated in ethyl alcohol, and washed in tap water. Antigen retrieval was carried out in 10 mM sodium citrate buffer for 20 min using a Black & Decker vegetable steamer. Primary antibodies for E-cadherin, Snail, α-SMA, and fibronectin were diluted to the appropriate concentrations with 2% casein in bovine serum albumin (BSA), and then were added to the slides with an overnight incubation at 4°C. After washing, a secondary antibody was added for 20 min, and the slides were washed and incubated with a tertiary PAP complex for 20 min. Diaminobenzidine was added for 2 min and the slides were counterstained with hematoxylin. A semi-quantitative score of staining intensity was determined by examining at least 5 fields of the peritoneum in each section under ×400 magnification and by digital image analysis (MetaMorph version 4.6r5, Universal Imaging Corp., Downingtown, PA, USA). For Masson's trichrome staining, 5 µm-thick sections of paraffin-embedded tissues were deparaffinized, hydrated in ethyl alcohol, washed in tap water, and re-fixed in Bouin's solution at 56°C for 1 hr. After washing in running tap water for 10 min and staining with Weigert's iron hematoxylin working solution for 10 min, the sections were stained with Biebrich scarlet-acid fuchsin solution for 15 min, followed by a 10-min wash. The slides were then differentiated in phosphomolybdic-phosphotungstic acid solution for 15 min, transferred to aniline blue solution and stained for 10 min, and were reacted with 1% acetic acid solution for 5 min. The thickness of the peritoneum, which was defined as the tissue between the mesothelial surface and the underlying muscle or parenchyma, was assessed as previously described [26]. Briefly, the maximal thickness of the peritoneum was measured in three Masson's trichrome-stained tissue sections per rat and five fields, the center of which included the area of maximal thickness, and were examined under ×400 magnification. Areas and perimeter lengths of the peritoneum were obtained from drawn outlines and the average thickness was calculated from rectangular approximation based on the values for area and perimeter in each field of view.

### Statistical analysis

All values are expressed as means ± standard errors of the mean (SEM). Statistical analyses were performed using the statistical package SPSS for Windows Ver. 11.0 (SPSS, Inc., Chicago, IL, USA). Results were analyzed using one-way ANOVA with a *post hoc* Bonferonni's test for multiple comparisons. P-values <0.05 were considered statistically significant.

## Results

### Effects of simvastatin on EMT and fibronectin expression in HPMCs

**Figure 1:**
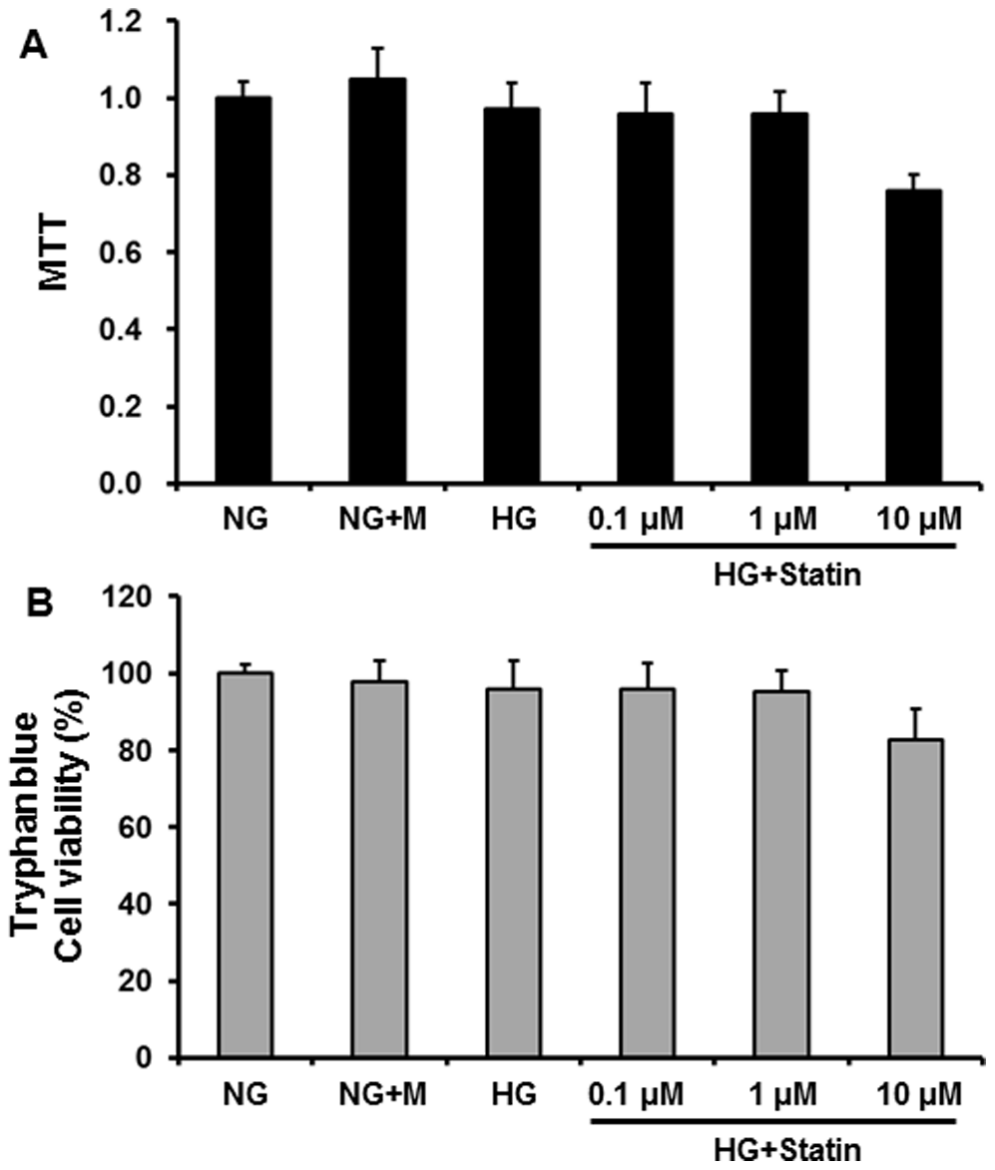
MTT (A) and tryphan blue (B) assay for cell viability. HPMCs were incubated for 72 hr with 5.6 mM glucose (NG), NG+ mannitol (94.4 mM, NG+M), high glucose (100 mM, HG), or HG+0.1 µM, 1 µM, or 10 µM simvastatin (HG + statin). Cell viability was maintained at up to 1 µM simvastatin, but was decreased by 20% at 10 µM. *; p<0.05 vs. other groups.

**Figure 2:**
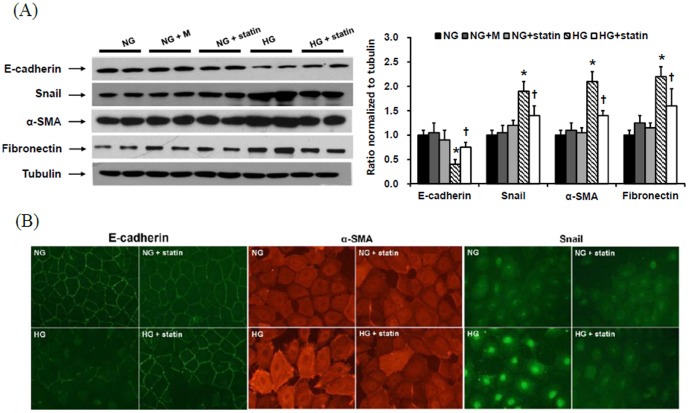
Effects of simvastatin on EMT and fibronectin expression in HPMCs. (A) HPMCs were incubated for 72 hr with 5.6 mM glucose (NG), NG + mannitol (94.4 mM, NG+M), NG+1 µM simvastatin (NG + statin), high glucose (100 mM, HG), or HG+1 µM simvastatin (HG + statin) (A representative of five Western blots). E-cadherin protein expression was significantly lower, while the protein expression of Snail, α-SMA, and fibronectin were significantly higher in HG-stimulated HPMCs compared to NG cells, and these changes were significantly attenuated by simvastatin. *; p<0.05 vs. NG, ^†^; p<0.05 vs. HG. (B) Compared to NG cells, HPMCs cultured under HG medium showed a weak staining of E-cadherin, a strong signal intensity of α-SMA, and increased nuclear translocation of Snail, all of which were ameliorated by the administration of simvastatin (×400).

As shown in Figure 1, the MTT assay demonstrated that HPMCs remained viable at up to 1 µM of simvastatin, but the viability was decreased by 20% at 10 µM. The viability was also assessed by trypan blue exclusion, in which all groups demonstrated>95% viability, suggesting no differences between control and simvastatin (1 µM)-treated groups. Therefore, the dose of 1 µM was used for the experiments. To evaluate the effects of statins on EMT *in vitro*, HPMCs were incubated for 72 hr with NG, NG+M, NG+simvastatin, or HG with or without simvastatin. E-cadherin protein expression was significantly lower, while the protein expression of Snail, α-SMA, and fibronectin were significantly higher in HG-stimulated HPMCs compared to NG cells (P<0.05) (Fig. 2A). Furthermore, the changes in HPMCs exposed to HG were significantly abrogated by simvastatin treatment (P<0.05) (Fig. 2A). These findings were corroborated by the immunofluorescence analysis. HPMCs cultured under HG medium showed a weak staining of E-cadherin, a strong signal intensity of α-SMA, and increased nuclear translocation of Snail, all of which were ameliorated by the administration of simvastatin (Fig. 2B). On the other hand, mannitol used as an osmotic control had no effect on EMT and fibronectin expression in HPMCs. In addition, the expression of EMT markers and fibronectin in NG cells was not affected by simvastatin.

**Figure 3:**
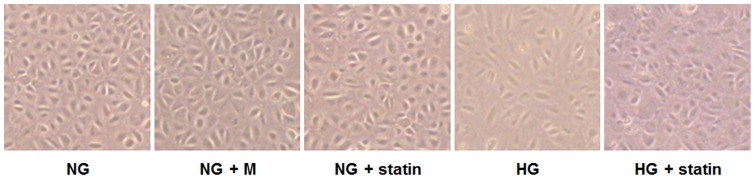
Morphologic changes under an inverted phase-contrast microscope in HPMCs exposed to 5.6 mM glucose (NG), NG + mannitol (94.4 mM, NG+M), NG+1 µM simvastatin (NG + statin), high glucose (100 mM, HG), or HG+1 µM simvastatin (HG + statin). The cobblestone-like appearance of HPMCs was converted into a fibroblast-like morphology 72 hr after HG treatment, which was reversed by simvastatin (×40).

Moreover, we observed the morphologic changes of HPMCs under an inverted phase-contrast microscope. The cobblestone-like appearance of HPMCs was converted into a fibroblast-like morphology after HG treatment, which was reversed by simvastatin (Fig. 3).

#### Activation of small GTPases such as RhoA and Rac1 in HPMCs

**Figure 4:**
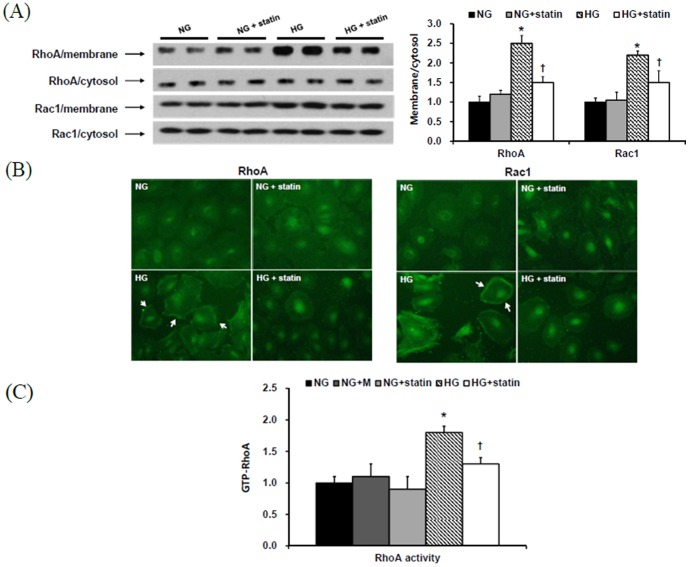
RhoA1 and Rac1 protein expression in the membrane and cytosol fractions of HPMCs exposed to 5.6 mM glucose (NG), NG + mannitol (94.4 mM, NG+M), NG+1 µM simvastatin (NG + statin), high glucose (100 mM, HG), or HG+1 µM simvastatin (HG + statin). (A) The protein expression of RhoA and Rac1 were significantly increased in the membrane fraction of HG-stimulated HPMCs compared to NG cells, and simvastatin significantly attenuated the increases in RhoA and Rac1 expression in the membrane fraction of HPMCs exposed to HG. *; p<0.05 vs. NG, ^†^; p<0.05 vs. HG. (B) An immunofluorescence study revealed that HG provoked the translocation of RhoA and Rac1 from the cytosol to the membrane fraction, and simvastatin treatment inhibited this translocation of RhoA and Rac1 induced by HG (×40). (C) The levels of Rho kinase were significantly increased in HG-treated HPMCs than in NG cells, and these changes were significantly abrogated by simvastatin. *; p<0.05 vs. NG, ^†^; p<0.05 vs. HG.

Posttranslational modification of Rho proteins by geranyl-geranlyation is essential for their membrane location and activity. Thus, the assessment of these proteins in the membrane fraction of the cells can reflect their degree of prenylation through the mevalonate pathway [27], [28]. Since inhibiting isoprenylation of the mevalonate pathway products was the main mechanism of statins, the membrane-associated protein expression of RhoA and Rac1 was evaluated in HPMCs by Western blot analysis after separation of the membrane and cytosol fractions. Compared to NG cells, the protein expression of RhoA and Rac1 were significantly increased in the membrane fraction of HG-stimulated HPMCs (P<0.05), and simvastatin significantly abrogated the increases in RhoA and Rac1 expression in the membrane fraction of HPMCs exposed to HG (P<0.05) (Fig. 4A). Furthermore, the immunofluorescence study revealed that HG provoked the translocation of RhoA and Rac1 from the cytosol to the membrane fraction, and simvastatin treatment inhibited this translocation of RhoA and Rac1 induced by HG (Fig. 4B). In addition, the levels of Rho kinase were significantly increased in HG-treated HPMCs than in NG cells (P<0.05), and these changes were significantly ameliorated by simvastatin (P<0.05) (Fig. 4C).

### Involvement of isoprenoids of the mevalonate pathway in EMT of HPMCs

**Figure 5:**
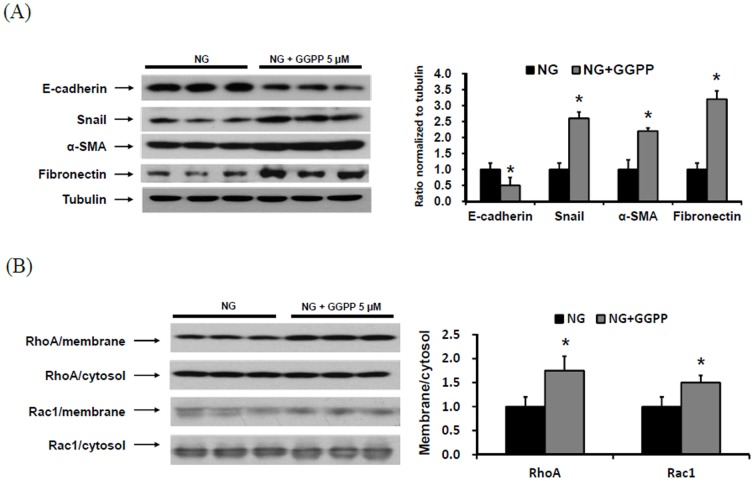
The protein expression of EMT markers and fibronectin in HPMCs. HPMCs were incubated with 5.6 mM glucose (NG) and NG+5 µM GGPP (NG+GGPP) for 72 hr. (A) GGPP treatment significantly decreased E-cadherin expression and significantly increased the protein expression of Snail, α-SMA, and fibronectin in HPMCs (A representative of five Western blots). *; p<0.05 vs. NG. (B) The protein expression of RhoA and Rac1 were significantly increased in the membrane fraction of HPMCs exposed to GGPP. *; p<0.05 vs. NG.

To evaluate whether isoprenoids of the mevalonate pathway were involved in peritoneal EMT, HPMCs were incubated with 5 µM GGPP for 72 hr. The administration of GGPP significantly decreased E-cadherin protein expression and significantly increased the protein expression of Snail, α-SMA, and fibronectin in HPMCs (P<0.05) (Fig. 5A). The protein expression of RhoA and Rac1 were also significantly increased in the membrane fraction of HPMCs exposed to GGPP (P<0.05) (Fig. 5B).

### Effect of small GTPase inhibitors on EMT and fibronectin expression in HPMCs

**Figure 6:**
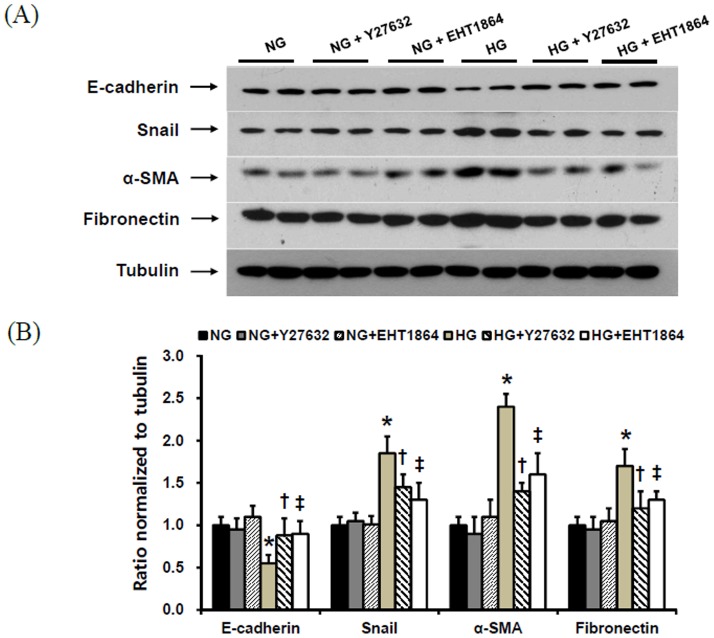
The protein expression of EMT markers and fibronectin in HPMCs exposed to 5.6 mM glucose (NG), NG with 1 µM Y27632 (Rho/ROCK inhibitor) (NG+Y27632), NG with 1 µM EHT1864 (Rac inhibitor) (NG+EHT1864), high glucose (100 mM, HG), HG with 1 µM Y27632 (HG+Y27632), or HG with 1 µM EHT1864 (HG+EHT1864). The administration of Y27632 and EHT1864 significantly attenuated the changes in EMT markers and fibronectin expression in HPMCs cultured under HG medium. *; p<0.05 vs. NG, ^†^; p<0.05 vs. HG, ^‡^; p<0.05 vs. HG.

Rho/ROCK inhibitor (Y27632) and Rac inhibitor (EHT1864) were added to HG-stimulated HPMCs, and the changes in EMT markers and fibronectin expression were determined. The administration of these two small GTPase inhibitors significantly ameliorated the changes in EMT markers and fibronectin expression in HPMCs cultured under HG medium (P<0.05) (Fig. 6).

### Effects of simvastatin on peritoneal EMT and ECM accumulation in a PD rat model

**Figure 7:**
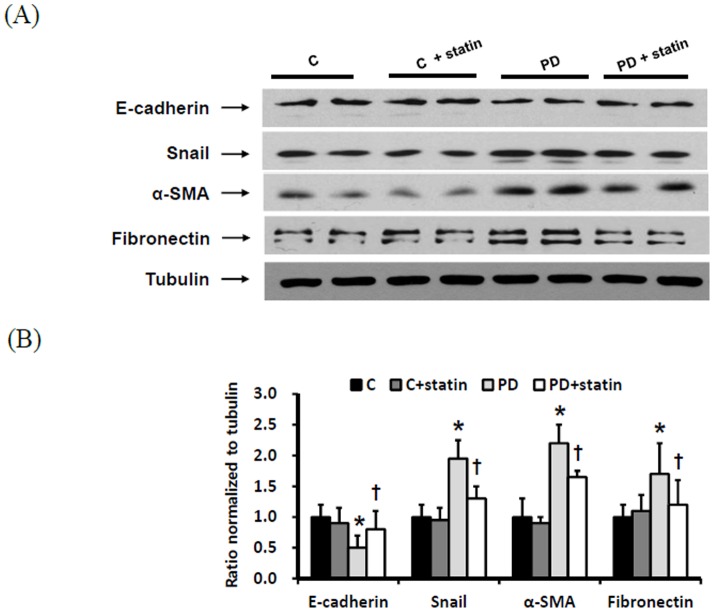
The protein expression of EMT markers and ECM in the peritoneum of control (C), C+ simvastatin (C + statin), 4.25% PDF instillation (PD), or 4.25% PDF + simvastatin (PD + statin) rats. E-cadherin protein expression was significantly lower, while Snail, α-SMA, and fibronectin protein expression were significantly higher in rats treated with 4.25% PDF compared to control rats, and these changes were significantly ameliorated by simvastatin. *; p<0.05 vs. C, ^†^; p<0.05 vs. PD.

**Figure 8:**
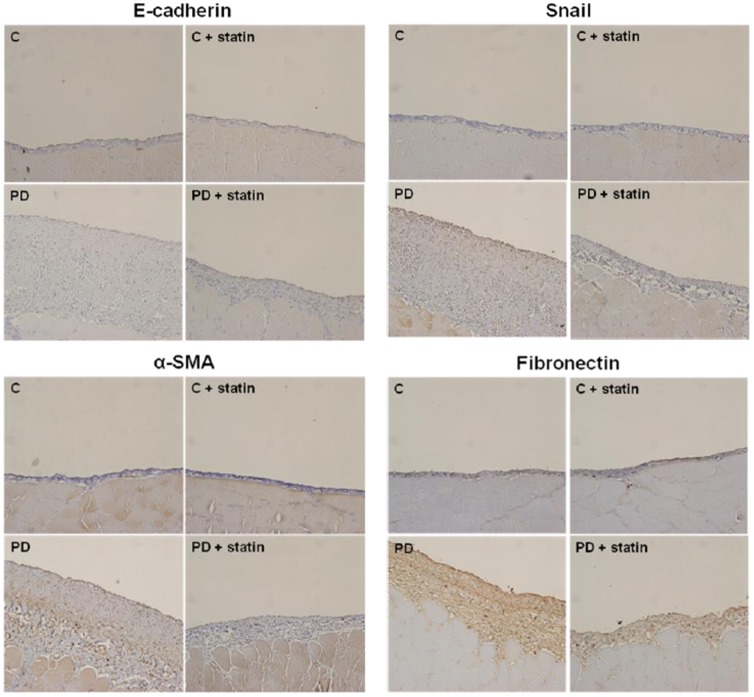
Immunohistochemical staining of the peritoneum of control (C), C+ simvastatin (C + statin), 4.25% PDF instillation (PD), or 4.25% PDF + simvastatin (PD + statin) rats. The intensity of E-cadherin staining was significantly lower, while Snail, α-SMA, and fibronectin staining intensities were significantly higher in PD rats compared to C rats, and simvastatin significantly ameliorated these changes in PD rats (×200).

**Figure 9:**
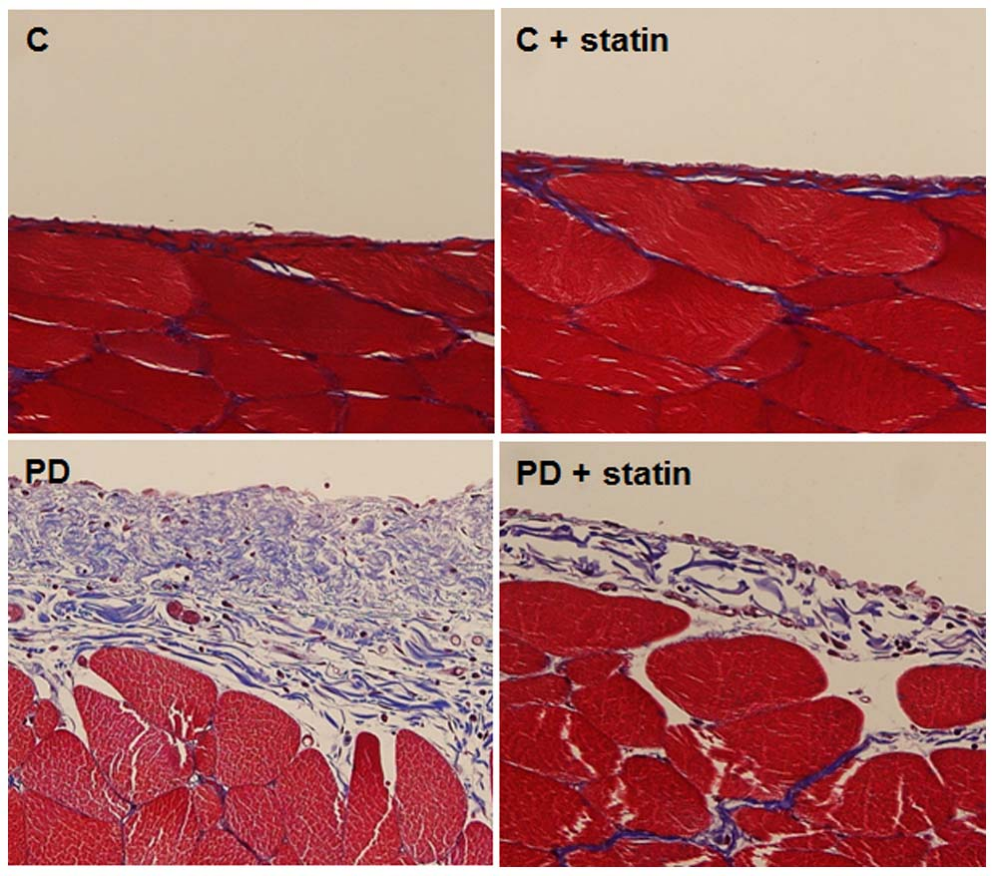
Masson's trichrome staining of the peritoneum of control (C), C+ simvastatin (C + statin), 4.25% PDF instillation (PD), or 4.25% PDF + simvastatin (PD + statin) rats. Peritoneal fibrosis assessed by Masson's trichrome staining was significantly more extensive in PD rats with 4.25% PDF than C rats, and these changes were significantly attenuated by simvastatin treatment (×200).

Finally, the effects of simvastatin on peritoneal EMT and ECM accumulation were explored in a PD rat model. E-cadherin protein expression was significantly lower, while Snail, α-SMA, and fibronectin protein expression were significantly higher in rats treated with 4.25% PDF compared to control rats (P<0.01), and these changes were significantly abrogated by simvastatin treatment (P<0.05) (Fig. 7). Immunohistochemical staining of the peritoneum also revealed that EMT markers and fibronectin protein expression were significantly higher in rats treated with 4.25% PDF relative to control rats, and simvastatin significantly attenuated EMT and ECM accumulation in PD rats (Fig. 8). Moreover, Masson's trichrome staining found that the submesothelial layer was significantly thicker and peritoneal fibrosis was more extensive in PD rats with 4.25% PDF than control rats, and these changes were significantly abrogated by simvastatin treatment (Fig. 9).

## Discussion

PF is one of the most serious complications of long-term PD, leading to membrane failure. Even though resident peritoneal fibroblasts and infiltrating inflammatory cells have been considered to play a key role in the development PF, EMT of PMCs has recently been highlighted as another potential mechanism responsible for PF [5]–[8]. This study shows for the first time that statin treatment abrogates PD-related EMT of HPMCs and ECM accumulation in a PD rat model. In addition, we demonstrate that these beneficial effects of statin are mediated, at least in part, by inhibiting isoprenylation of small RhoGTPases such as RhoA and Rac1.

Besides a physiologic role of EMT in embryogenesis or organ development, it also plays a pathologic role in cancer metastasis and fibrotic disorders.^7^ A number of recent studies have found that PMCs also undergo EMT during PD [5], [29]–[31]. In particular, Yanez-Mo *et al.* [5] showed that PMCs undergo a transition from an epithelial phenotype to a mesenchymal phenotype soon after PD is initiated and this process is accompanied by a decrease in the expression of cytokeratins and E-cadherin, suggesting that these cells indeed acquire structural changes during PD. Consistent with these findings, in the present study, E-cadherin expression was significantly decreased, while Snail, a-SMA, and fibronectin expression was significantly increased in HPMCs exposed to HG and in the peritoneum of rats instilled with 4.25% PDF. Furthermore, the cobblestone-like appearance of normal HPMCs was converted into a fibroblast-like morphology after HG treatment. These findings support previous evidence of EMT of PMCs under pathologic conditions.

HMG-CoA reductase inhibitors, or statins, are potent inhibitors of cholesterol biosynthesis and have emerged as the leading lipid-lowering agents. However, it has been acknowledged that the beneficial effects of statins are not mediated solely by their lipid-lowering property, but also through distinct “pleiotropic” effects [9]–[11]. In fact, statins exert these effects by preventing the synthesis of other important isoprenoids of the cholesterol biosynthetic pathway, such as FPP and GGPP that are downstream of the mevalonate pathway [12]. These intermediates play key roles in the post-translational modification of many proteins, including small GTP binding proteins—the family of Ras, Rho, Rap, and Rab GTPase—by serving as lipid attachments through a process known as “prenylation” [13]–[15]. Isoprenylation of these proteins permits the covalent attachment, subcellular localization, and intracellular trafficking of membrane-associated proteins [13]–[15]. Therefore, small G proteins are anchored to the cell membrane if they are prenylated, while they remain in the cytoplasm when prenylation is inhibited. In general, modification of FPP is necessary for localization of Ras, whereas GGPP is required for Rho, Rap, and Rab family proteins [12]. By inhibiting the synthesis of mevalonate products, statins prevent isoprenylation of small GTPases, leading to suppression of these signal molecules [14]. To support this notion, we clearly demonstrated that the expression of RhoA and Rac1 protein in the membrane fraction was increased in cultured HPMCs exposed to HG, and these increases were ameliorated by statin treatment.

In addition to the pivotal role of small RhoGTPases in the regulation of cell shape, adhesion, migration, secretion, and proliferation [12]–[14], several recent studies have found that small RhoGTPases such as RhoA, Rac1, and Cdc42 exert a direct effect on EMT in a number of cell types including renal [16]–[20], lens [32], bronchial [33], and mammary epithelial cells [34]. Bhowmick *et al.* [34] showed that a RhoA-dependent mechanism was responsible for TGF-β1-induced mammary epithelial EMT. Moreover, some investigators demonstrated that overexpression of active RhoA reduced E-cadherin expression and increased mesenchymal cell markers [16], while transfection of a RhoA dominant-negative vector or ROCK inhibition with Y-27632 or fasudil inhibited EMT provoked by angiotensin II in renal tubular epithelial cells [19]. This indicates that RhoA may be directly involved in renal tubular epithelial EMT. Furthermore, in contrast to the putative roles of Rac1 and Cdc42, which are believed to be involved in the establishment and maintenance of epithelial intercellular adhesions [35]–[37], activation of these proteins can also induce EMT accompanied by breakdown of cell-cell adhesion and rearrangement of the actin cytoskeleton [38]–[40]. Similar to these cells, EMT-like changes caused by small GTPases can occur in PMCs. A recent study by Zhang *et al.* [20] found that activation of RhoA in rat PMCs by TGF-β1 up-regulated α-SMA, vimentin, and collagen expression and down-regulated E-cadherin expression, suggesting that the RhoA/ROCK signaling pathway mediated EMT in rat PMCs in response to TGF-β1. Based on these findings, it is surmised that small GTPases such as RhoA, Cdc42, and Rac1 may be involved in EMT.

Because statins have an inhibitory effect on the synthesis of isoprenoid intermediates, it can be presumed that statins may reverse EMT-like changes through inhibiting isoprenylation of small RhoGTPases. This assumption was verified in the current study. We showed for the first time that statin treatment attenuated HG- or PD-induced EMT and ECM accumulation in HPMCs *in vitro* and *in vivo*. In addition, we provided an underlying mechanism of the effect of statins against EMT. The present study revealed that HG increased membrane translocation of RhoA and Rac1 and enhanced Rho-kinase activity in cultured HPMCs. Moreover, HG-induced changes in EMT markers were reversed by Rho and Rac inhibitors. Taken together, the results suggest that HG increased isoprenylation of small GTPases, and these proteins play a role in HG-induced EMT of HPMCs. Furthermore, GGPP-treated HPMCs lost epithelial markers and acquired mesenchymal markers, indicating that isoprenoid intermediates were directly involved in EMT of HPMCs. All these findings support evidence that statins can inhibit HG-induced EMT in HPMCs, at least in part, through inhibiting isoprenylation and subsequently inactivating RhoA and Rac1.

Even though this study underscores an important role of statins in terms of inhibiting small GTPases, it is possible that statins may exhibit this favorable effect via other mechanisms. In fact, EMT can be induced by a variety of cytokines or growth factors including TGF-β [30], angiotensin II [41], fibroblast growth factor-2 [42], epidermal growth factor [43], and platelet-derived growth factor [44]. Furthermore, it can be triggered by inflammation or oxidative stress [45], and statins have been reported to abrogate some of these stimuli such as inflammation, oxidative stress [46]–[48], connective tissue growth factor [49], or TGF-β [50]. However, there is a lack of evidence supporting a role for statins in inhibiting EMT by reducing these triggering factors. On the other hand, our *in vivo* experiment demonstrated that alteration of EMT markers and increased ECM accumulation in a PD rat model were not completely ameliorated by statin treatment. Based on these findings, it is implied that peritoneal EMT is a complex process which is engaged by a wide spectrum of factors other than RhoA and Rac1 activation. Therefore, the results of the current study should be interpreted with caution, but provide another potential mechanism of the pleiotropic effects of statins with respect to inhibiting EMT. Finally, it is possible that beneficial effect of statins is mediated through its fibrinolytic activity [51], [52]. Therefore, we measured tissue-type plasminogen activator (t-PA) concentration in cell-conditioned media by ELISA and determined plasminogen activator inhibitor-1 (PAI-1) expression by Western blot analysis. Interestingly, t-PA level was significantly increased in HG-treated cell media compared to controls, which was not altered by statin treatment. On the other hand, PAI-1 expression was significantly increased in HG-treated cells, while statin treatment decreased the increased expression of PAI-1 in these cells (data not shown). Although t-PA has been shown to play a role in fibrinolysis, it is reported to destruct tubule-epithelial basement membrane, which promotes EMT [53]. Thus, the increased t-PA level by HG treatment can be interpreted as a process of EMT. In fact, our finding is supported by several studies suggesting increased t-PA level in HG-treated vascular endothelial cells [54] and PDF-treated HPMCs [55]. Of note, we also found that statin treatment did not further alter the increased t-PA level in HG-treated cells. This finding is not consistent with previous studies showing that statins increase t-PA activity [51], [52] Such discrepancy is likely due to differences in cell type, time of simulation, dose, and type of statins. Nevertheless, PAI-1 expression was significantly decreased by statin treatment. Although our finding cannot fully support fibrinolytic activity of statin, it suggests that statins can maintain balance between t-PA and PAI-1, thus resulting in collagen degradation. This finding adds more strengths of statin in terms of preserving peritoneal membrane in addition to attenuating EMT.

In conclusion, the present study found that PD-related EMT was mediated through isoprenylation and subsequently activation of RhoA and Rac1 in mevalonate pathway and statin treatment attenuated EMT changes in HG-stimulated HPMCs and 4.25%-PDF-instilled PD rats. These findings suggest that statins may be a promising therapeutic strategy for preservation of peritoneal membrane integrity in long-term PD patients.
